# CpG content-dependent associations between transcription factors and histone modifications

**DOI:** 10.1371/journal.pone.0249985

**Published:** 2021-04-15

**Authors:** Jonas Fischer, Fatemeh Behjati Ardakani, Kathrin Kattler, Jörn Walter, Marcel H. Schulz

**Affiliations:** 1 Max Planck Institute for Informatics, Databases and Information Systems, Saarbrücken, Germany; 2 Max Planck Institute for Informatics, Computational Biology and Applied Algorithmics, Saarbrücken, Germany; 3 Cluster of Excellence for Multimodal Computing and Interaction, High Throughput Genomics and Systems Biology, Saarbrücken, Germany; 4 Department of Genetics, University of Saarland, Saarbrücken, Germany; 5 Institute of Cardiovascular Regeneration, Goethe University, Frankfurt, Germany; Università degli Studi di Milano, ITALY

## Abstract

Understanding the factors that underlie the epigenetic regulation of genes is crucial to understand the gene regulatory machinery as a whole. Several experimental and computational studies examined the relationship between different factors involved. Here we investigate the relationship between transcription factors (TFs) and histone modifications (HMs), based on ChIP-seq data in cell lines. As it was shown that gene regulation by TFs differs depending on the CpG class of a promoter, we study the impact of the CpG content in promoters on the associations between TFs and HMs. We suggest an approach based on sparse linear regression models to infer associations between TFs and HMs with respect to CpG content. A study of the partial correlation of HMs for the two classes of high and low CpG content reveals possible CpG dependence and potential candidates for confounding factors in our models. We show that the models are accurate, inferred associations reflect known biological relationships, and we give new insight into associations with respect to CpG content. Moreover, analysis of a ChIP-seq dataset in HepG2 cells of the HM H3K122ac, an HM about little is known, reveals novel TF associations and supports a previously established link to active transcription.

## Introduction

Epigenetic modifications play a crucial role in the gene regulatory system. They allow a single DNA molecule to be interpreted in different cell types and throughout different developmental stages, by directly interacting with other regulatory factors or by reorganizing the DNA accessibility in the cell and thereby affecting gene transcription [1]. The genomic location of certain epigenetic modifications, the post-translational modifications of histone residues, can be measured by genome-wide assays such as chromatin immunoprecipitation followed by massive parallel sequencing (ChIP-seq) [2, 3]. Several consortia such as ENCODE [4, 5] and IHEC [6] have surveyed many of these modifications for a number of cell types and cell lines. With this data at hand, epigenetics has the potential for a better understanding of, for instance, the cause and effect of diseases [7–10] and can lead to the development of novel therapeutic approaches for cancer [11, 12] or HIV [13].

Here, we consider the interplay between two important layers of gene regulation, DNA-binding transcription factors (TFs), and post-translational modification of histone residues (HMs) of the nucleosome complex. Co-localization and interactions between both these layers have been observed and have proven to be useful for determining the expression level of nearby genes [14–19]. Approaches to model the chromatin signaling network using HMs and chromatin modifying enzymes, including certain TFs, yielded novel insights into interactions between these components in the gene regulatory network [20]. However, how precisely TFs and HMs interact with each other remains unclear and many facets, such as affiliation to protein families [21], sequence composition [22], or feedback loops [23], appear to be relevant for the complex regulatory machinery. Recent work suggested that the information provided by TFs and HMs jointly is redundant in predicting gene expression [24, 25], suggesting that these two entities are tightly coupled. This is in line with previous findings that showed the ability of certain TFs to recruit histone modifying enzymes [26], and that certain HMs amplify the likeliness of TFs binding to specific genomic positions [27]. Furthermore, direct physical contacts of HMs and TFs have been reported previously [28], as well as destabilizing properties of HMs for heterochromatic regions, which make these regions generally more accessible for proteins [29].

In the past, different computational models have been proposed to elucidate the role of Epigenetic factors in gene regulatory networks and, in particular, to discover patterns and associations from epigenetic data about TF binding and HM occurrence. From the data perspective, the models can be categorized into three different groups, 1) unsupervised approaches using TF and HM data, 2) the prediction of TF binding based on HM data, and 3) the prediction of HM binding based on TF data.

Successful unsupervised approaches leveraged graphical models for discovering interactions between TF and HMs. Lasserre et al. [30] combined HM ChIP-seq data with mRNA and DNase hypersensitivity information to infer an undirected interaction network using sparse partial correlation, which was able to recover many known interactions. In another, but related study, the authors of ChromNet derived group graphical models based on inverse correlation of ChIP-seq data, including TFs and HMs [31]. For the prediction of TFs based on HM data, one approach derived TF binding affinities from HM data using multiple linear regression and random forests, focusing on the discovery of differential epigenetic patterns between promoter and distal regions in the genome [32].

For the purpose of predicting HM information based on TF data, different methods have been employed. Whitaker et al. use DNA binding motifs to make a binary prediction of the presence or absence of an HM peak employing a LASSO logistic regression model to filter predictive motifs and a Random Forest on the filtered motifs to classify whether an HM is present at a specific region [33]. Benveniste et al. tackle the similar problem of predicting the HM existence in a window of 100bp along the Transcription Start Site (TSS). However, instead of using the TF motifs as in Whitaker et al. [33], they estimated the abundance of TF ChIP-seq data obtained from ENCODE [34]. Perner et al. later combined partial correlation with an elastic net based regression approach to infer associations between a small set of chromatin modifiers and HMs [20]. Their method leverages the potential of regression based approaches combined with the ChIP-seq data available in databases to gain insight into the interplay of TFs and HMs.

In our work we build up on the previous approaches by extending the existing ideas to capture more complex relationships, investigating the impact of CpG content on the regulatory machinery, and studying new data. In contrast to Benveniste et al., we use a LASSO regression based approach to be able to investigate negative associations between TFs and HMs and to use the coefficients as a mean to measure the strength of their association. Furthermore, a LASSO approach implicitly selects a subset of TFs that impact HM occupancy, by driving other coefficients to zero. This implicit feature selection allows us to distill the factors relevant for HMs from a large set of TFs without prior knowledge. We extend the work of Perner et al. by using a larger set of TFs, not restricting to chromatin modifiers, to be able to derive novel, unknown interactions, or associations between TFs and HMs. Furthermore, we binned the region around the TSS into windows of 100bp length to improve resolution and added a fusion penalty to enforce similar coefficients for adjacent bins in order to better resemble the input signal. Apart from these technical extensions, our main contribution lies in the analysis of the impact of promoter CpG content on the derived models by splitting the gene set into promoters with high and low CpG content, following the ideas of Karlic et al., and Saxonov et al. [22, 35]. It was previously shown that different histone marks are associated with gene expression in CpG-rich versus CpG-poor promoters [29, 35] and that CpG-poor promoters are enriched in binding sites of tissue-specific TFs [36]. Thus we speculate that TF–HM associations may differ in these two promoter classes. Furthermore, we use our method to study associations of TFs to H3K122ac, a mark that only recently draw attention as first reports show an association with active gene expression [37].

## Materials and methods

### Basic model

**Fig 1:**
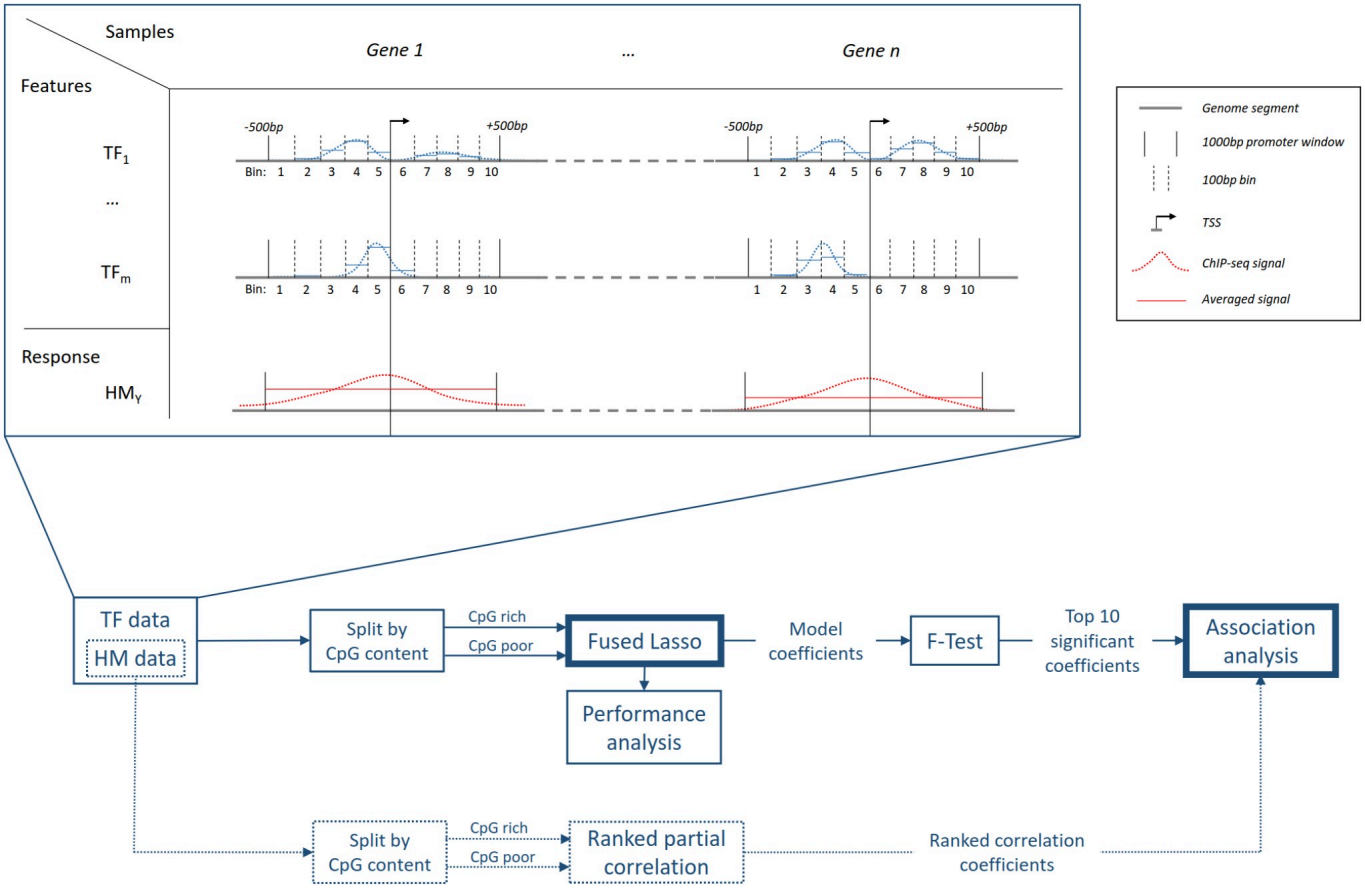
Workflow of this association study. **Top**: Summary of the data processing pipeline. Promoter regions of protein coding genes are taken as samples (columns). Each HM ChIP-seq signal (dotted red lines) in a window (black vertical lines) from 500bp upstream to 500bp downstream of the TSS is averaged to obtain the response values for our models (solid red lines). For the TF data, we further segment this window into ten non-overlapping bins (black vertical dashed lines), each of which is of length 100bp. The TF ChIP-seq signal (blue dotted lines) is averaged (solid blue lines) inside each bin to obtain a higher resolution for the features. **Bottom**: Sketch of the steps involved in this association study. Starting from the data set, described at the top, the samples (gene sets) are split by CpG content and separate models for each set are trained. The derived coefficients are then processed to test for statistical significance with an F-Test and the top 10 significant coefficients are used for the association analysis. To further support the analysis, we examine the ranked partial correlation coefficients of the Histone Modification data.

HM presence has been associated with the presence of several TFs [20, 31, 34]. Thus, it is suggestive to predict the presence of a given HM from a set of TFs that could recruit other histone modifying enzymes or directly act as readers, writers, or erasers of that HM. A straightforward way of predicting HMs based on TFs is to train a regression model that takes the abundance of *m* TFs as input (features) and the abundance of an HM as output (response). Thus, we can formulate the prediction of the abundance abd(HM_*Y*_) of a Histone Modification HM_*Y*_ as

$$\begin{array}{c} \text{abd}\left({\text{HM}}_{Y}\right)\approx \beta_{0}+\beta_{1}\times \text{abd}\left({\text{TF}}_{1}\right)+\beta_{2}\times \text{abd}\left({\text{TF}}_{2}\right)+\cdots +\beta_{m}\times \text{abd}\left({\text{TF}}_{m}\right), \end{array}$$ (1)

where *β*_0_ is the model bias term and *β*_*i*_, *i* = 1‥*n* are model coefficients subject to optimization. In our case, the abundance of both TFs and HMs is obtained by using ChIP-seq measurements in non-overlapping windows from 500bp upstream to 500bp downstream of the transcription start site (TSS) of all annotated protein coding genes. To get a more refined resolution of the ChIP-seq data for the features, we further bin those windows into segments of 100bp length, in which read counts of the TF ChIP-seq data are accumulated (Fig 1 top). To prevent a loss of prediction power due to outliers and differing signal strength, caused by e.g. measurement errors, noise, and distinct antibody affinity, we normalize the logarithm of each signal to [0, 1] (abd_*norm*_) instead of using the raw read counts. Thus, we modify Eq 1 to obtain the following model:

$$\begin{array}{c} \text{abd}\left({\text{HM}}_{Y}\right)\approx \beta_{0}+\beta_{1,1}\times {\text{abd}}_{norm}\left({\text{TF}}_{1},{\text{Bin}}_{1}\right)+\beta_{1,2}\times {\text{abd}}_{norm}\left({\text{TF}}_{1},{\text{Bin}}_{2}\right)+\cdots +\beta_{m,k}\times {\text{abd}}_{norm}\left({\text{TF}}_{m},{\text{Bin}}_{k}\right), \end{array}$$

considering *k* bins for each of the *m* TFs. To gain more interpretability, we impose regularizations on the coefficients. First, we add an *L*_1_ norm of the coefficients as a penalization term to achieve sparsity, thus reducing the number of TFs selected for an HM to the ones with the strongest relationship to that HM. One approach to solve linear regression with *L*_1_ norm is LASSO and has been established in the machine learning community, known to recover features that are important to explain the response variable and setting feature coefficients which are weakly correlated with the response to zero [38]. Second, we use an additional penalty term for the absolute difference between coefficients of adjacent bins to maintain the biological signal among adjacent bins of a window. In addition to a sparsity constraint, the generalized fused LASSO [39, 40] incorporates such a fusion penalty, allowing the specification of a feature graph *G* = (*V*, *E*), such that for each pair of features (vertices) connected by an edge, a penalization for the absolute difference between their coefficients is added to the optimization function. In our case, *E* is the set of pairs of adjacent bins.

Thus, the final objective function that is optimized to find a set of coefficients $\hat{\beta }$ can be written in Lagrangian form as

$$\begin{array}{c} \hat{\beta }=\underset{\beta }{arg min}({\left\|(Y-\beta X)\right\|}_{2}^{2}\;+\;\lambda_{1}\left({\left\|\beta \right\|}_{1}^{1}\right)+\lambda_{2}\sum_{(x_{i},x_{j})\in E}{\left\|\beta_{i}-\beta_{j}\right\|}_{1}^{1}), \end{array}$$

where λ_1_ and λ_2_ are Lagrangian multipliers adjusting the contribution of the two penalization terms to the overall loss.

### Model training and assessment

To train the models we use the generalized fused LASSO implementation provided by Arnold et al. [40]. For a given data set, we performed a 5-fold nested cross validation, with parameter optimization in the inner folds based on RSS, and measured model performance in terms of RSS and Spearman correlation coefficient on the outer folds. The implementation expects one parameter $\gamma =\frac{\lambda_{1}}{\lambda_{2}}$, where *γ* = 0 applies only fusion and *γ* = 1 enforces similar sparsity and fusion penalties in the model. The parameter *γ* is explored within the set of values {0} ∪ {10^*i*^|*i* = −5, −4, ⋯, 5}. The λ_2_ values corresponding to the knots at the solution path are then derived for each *γ* value.

### Inference of associations

For each of the models, we examined the impact of each TF on the response signal of an HM. For this, we summed the coefficients over all bins of a TF and performed an F-test with significance cutoff *α* = 0.005 to assess the statistical significance of the derived coefficients. We computed the F-statistic as

$$\begin{array}{c} F=\frac{{\text{RSS}}_{0}-{\text{RSS}}_{\text{full}}}{\text{df}\left(M_{0}\right)-\text{df}\left(M_{\text{full}}\right)}\times \frac{N-\text{df}(M_{\text{full}})-1}{{\text{RSS}}_{\text{full}}}, \end{array}$$

where RSS_0_ denotes the residual sum of squares for the reduced model by excluding the TF we test for, RSS_full_ is the RSS of the full model [38]. The variables $\text{df}(M_{0})$ and $\text{df}(M_{\text{full}})$ are the degrees of freedom (df) of the reduced and full model, respectively. Due to the fusion penalty in the objective function, the df of fused features are counted as one. Thus, the df of the model are computed as the number of nonzero blocks of coefficients, where one block is a set of fused coefficients having the same value. This definition of the degrees of freedom is suggested by the authors of fused LASSO [39]. As re-training the model for each TF removed would be intractable, we compute the RSS of each reduced model (RSS_0_) by setting the coefficients of the TF we test for to zero to estimate the F-statistic.

Once we computed p-values for each TF, we report the TFs with the ten lowest p-values that are smaller than *α* as important and significant. This ensures that, on the one hand all TFs that we consider are statistically significant for predicting the response, but on the other hand maintains a comprehensible subset of TFs. The overall workflow of our approach is depicted in Fig 1.

### CpG content based set partitions

**Fig 2:**
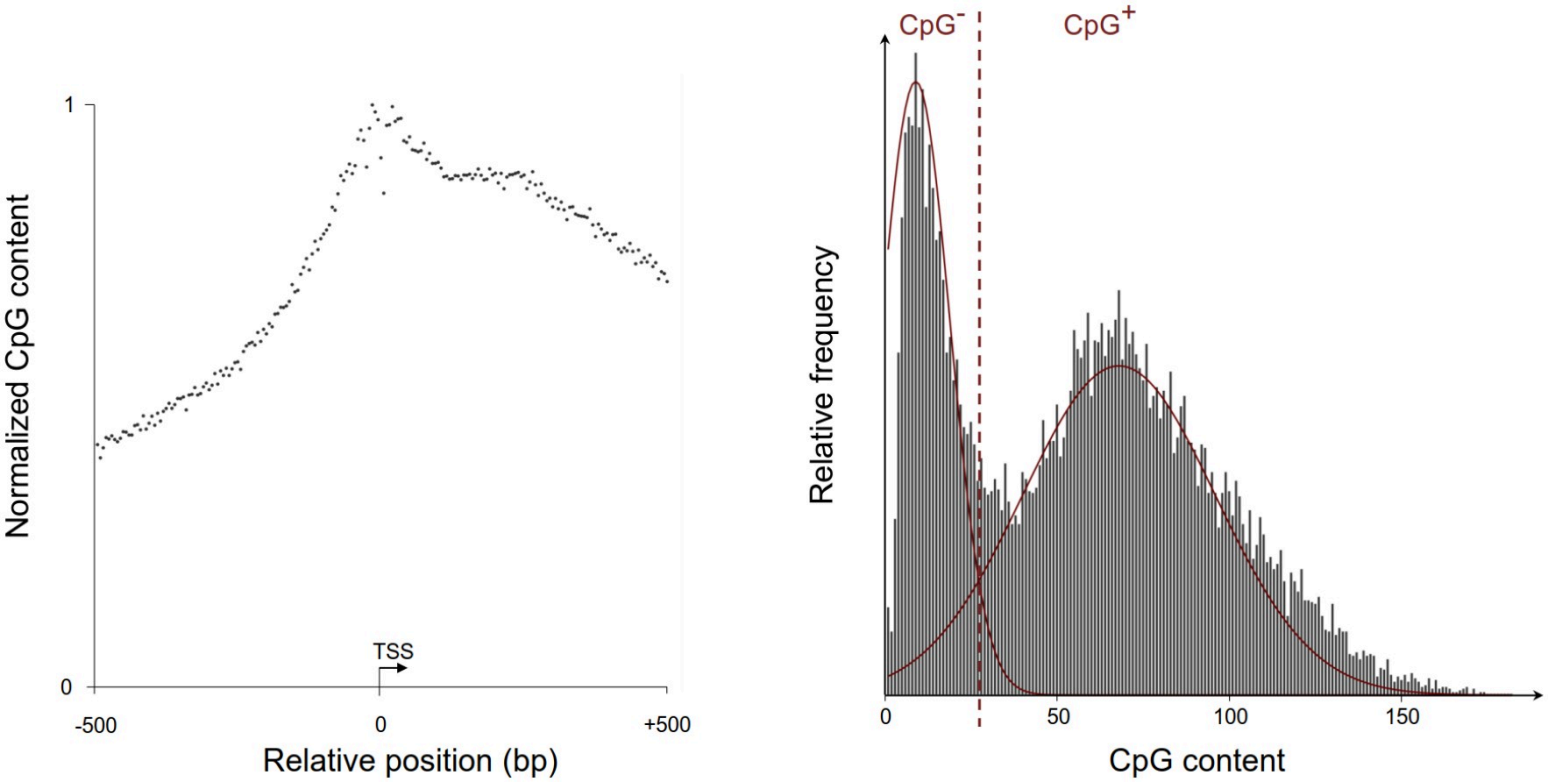
*CpG distribution around TSS* Visualized are the distributions of CpGs from 500bp upstream to 500bp downstream of TSSs of protein coding genes of the hg19 reference in bins of width 5. **Left**: Relative CpG distribution along the gene regions of interest for this work. **Right**: Histogram of CpG content of genes, summed over all bins per gene, with Gaussians indicated for the two modes of the distribution. The intersection of the Gaussians defines the split into CpG poor (left side) and CpG rich genes (right) indicated by the dashed red line.

It is known that CpG content has an impact on different components of the gene regulatory system [41, 42]. For example, most housekeeping genes show CpG enriched promoter regions [43] whereas tissue-specific genes most frequently harbor CpG poor promoters [36]. Besides, it is evident that CpG content in promoter regions spikes around the TSS, decreasing with growing distance from the TSS (compare Fig 2).

To investigate the influence of genomic CpG content on the relationship of TFs and HMs, we partition the promoter regions of protein coding genes into those with high, and those with low CpG content for the given reference genome. We thus obtain a criterion that allows us to split the samples of each experiment into two groups, that we can model separately to reveal CpG dependent trends. This split into two groups does arise naturally from the promoter regions, as Saxonov et al. showed by looking at frequencies of CpG content across genes [22]. That revealed a clear bimodal distribution of CpG content that leads to two classes as well as a threshold separating the two modes.

Here, we reproduced their results for hg19, the reference genome build for the processed data, by counting the number of CpGs from 500bp upstream to 500bp downstream of TSSs of protein coding genes, counting CpGs in bins of width 5. This yields a bimodal distribution analogous to Saxonov et al. [22], which can be fitted by two Gaussians visualized in Fig 2b. The intersection of the two Gaussians gives a threshold to classify the CpG poor and CpG rich promoters. We end up with a split of samples into 3772 promoters with low, and 10240 promoters with high CpG content. We apply this split to each HM and TF ChIP-seq data set to train separate models for high, respectively low CpG content. All our models were trained on each partition as well as the whole data to be able to exclude any influence of training set size on the performance in the analysis of the individual models.

### Partial correlation analysis

**Fig 3:**
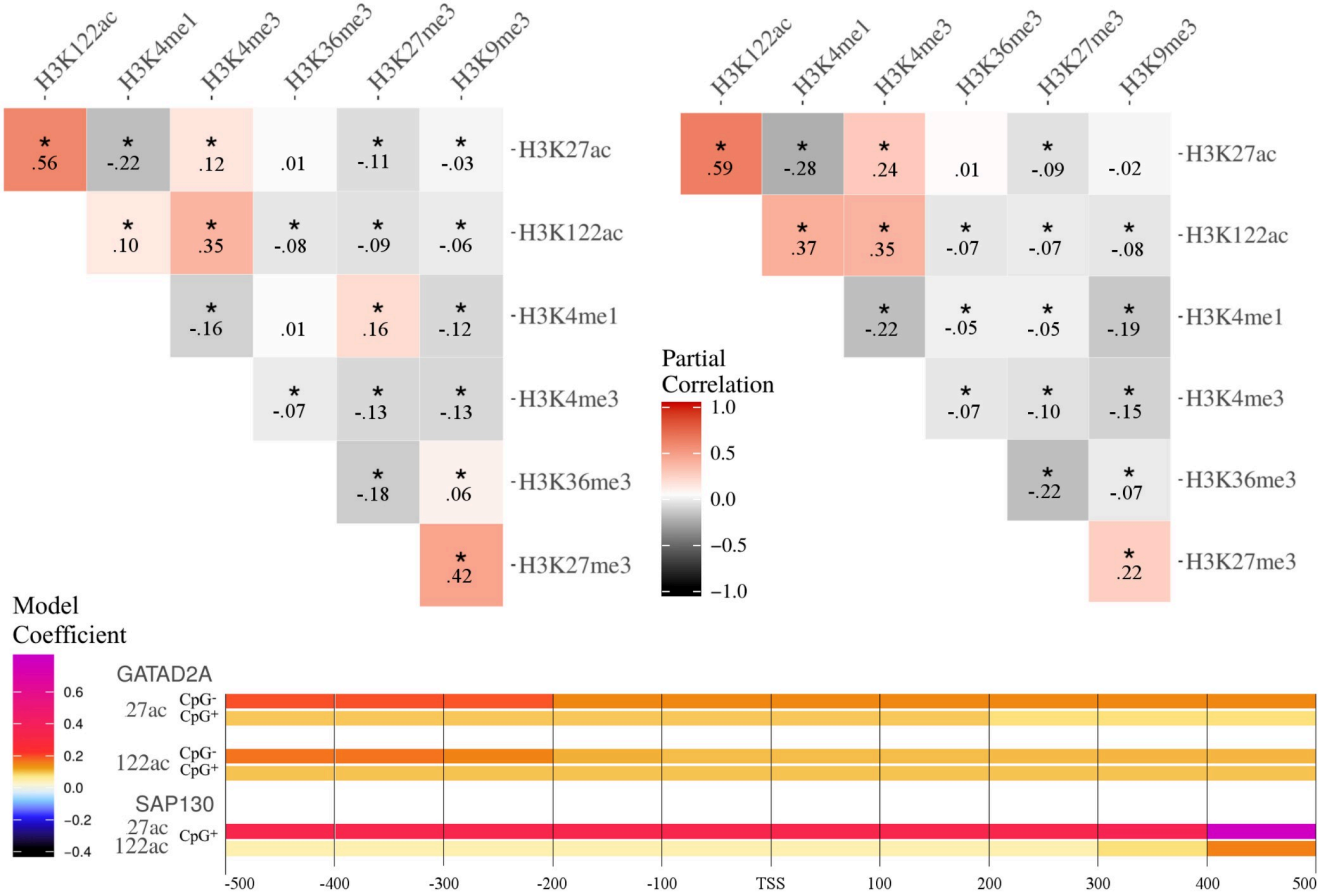
*Partial correlation and spatial coefficient analysis for HepG2* Top: Partial correlation heatmap between histone modifications in CpG rich (left) and poor (right) promoters in HepG2. Partial correlation coefficients are computed on mean ChIP-seq signal on the promoter regions of the gene sets. Significant partial correlations are marked with *. **Bottom**: For selected models, coefficients for two TFs that deviate strongly across bins are shown as a heatmap. Color scale for model coefficients is given on the left.

Partial correlation of two variables *X* and *Y* given a set of other variables Z, is computed as the Pearson correlation coefficient *ρ* between the residuals of the two linear regression models predicting *X* with Z and *Y* with Z, respectively. Thus, the partial correlation can be seen as the Pearson correlation of two variables *X* and *Y* after removing the information of other variables Z. In our study, we compute the Partial Correlation for each pair of HMs, separately for the CpG poor and rich promoter genes. To assess the significance of the correlations, we carry out a permutation test assuming that the observed Partial Correlations between *X* and *Y* given Z is generated by chance from the marginals of the residuals $r_{X|Z}$, $r_{Y|Z}$. Thus, we draw *n* = 10000 permutations of the residuals $r_{X|Z}^{\sigma }$ with *σ* indicating that these residuals are permuted and keep $r_{Y|Z}$ fixed, then computing the Partial Correlation based on the permuted residuals $\rho (r_{X|Z}^{\sigma },r_{Y|Z})$. The p-value is obtained as the number of observed Partial Correlations of the permuted residuals being larger than the actual Partial Correlation, divided by *n*, and a significance threshold of *α* = 0.01 is set for Bonferroni corrected p-values. The results are summarized in Fig 3, with significant Partial Correlations marked with an asterisk.

### Data

All models were generated on data of the immortalized human liver carcinoma cell line HepG2 and leukemia cell line K562. The HM data is a set of files derived from ChIP-Seq experiments produced within the DEEP project [44] representing the read counts mapped to regions over the whole human genome (reference genome version hg19). The experiments comprise the modifications H3K27me3, H3K27ac, H3K36me3, H3K4me1, H3K4me3 and H3K9me3. Additionally, an experiment of H3K122ac is available for the HepG2 cell line. The abundance of 158 and 111 TFs was measured by the ENCODE consortium using ChIP-Seq experiments for K562 and HepG2, respectively.

The HepG2 cell line HM data has been obtained through the DEEP project. This data has been deposited at the European Genome-Phenome Archive (EGA) under the accession number EGAS00001001937 (https://www.ebi.ac.uk/ega/home). For the K562 cell line both HM and TF, as well as HepG2 TF data can be accessed through the encode portal (https://www.encodeproject.org/matrix/?type = Experiment). S1 and S2 Tables in S1 File contain ENCODE accession IDs for the TF data obtained from the ENCODE portal, the former for the K562 cell line, the latter for HepG2. S3 Table in S1 File contains ENCODE accession IDs for HM data obtained for K562 from ENCODE.

## Results

In this section we present the results for our analysis of TF and HM ChIP-seq data in the light of CpG content in promoter regions. The analysis, for which the overview is depicted in Fig 1, is separated into two parts. The first part comprises a partial correlation analysis of the HM ChIP-seq signals, the second part involves the analysis of regularized regression models that allow to derive relationships between TFs and HMs from ChIP-seq data. The partial correlation analysis help our understanding of confounding relationships between TFs and HMs. Such confounding relationships are likely to appear as e.g. HMs that are linked to active transcription are likely to be co-located in active promoter regions. Thus, a discovered TF–HM relationship might be just spurious, if the TF is actually interacting with a co-located mark. Hence, we can leverage the knowledge from the partial correlation analysis for the interpretation of our regression models. Starting with insights from this partial correlation, we then present results that reveal CpG content dependence of regression model performance, promoter locality of TF–HM relationships, and show general relationships that reflect known interactions as well as potential new interaction partners. Finally, we present results on a new data set of H3K122ac.

### Partial correlation between HMs suggests potential CpG switches

For the partial correlation analysis, depicted in Fig 3, we can observe a strong partial correlation coefficient between H3K9me3 and H3K27me3, which are both associated with heterochromatin. Also, H3K36me3 and H3K9me3 show a high partial correlation coefficient value. Recent work reported the presence of H3K36me in constitutive heterochromatin, which is often marked with H3K9me3 [45]. Furthermore, we can see a weak positive correlation between H3K27ac and H3K4me3, both found in euchromatic regions. The marks H3K4me1 and H3K4me3 show a strong negative value, as these HMs are mutually exclusive as they appear on the same residue. For H3K122 we can observe a strong partial correlation with both H3K27ac and H3K4me3. Intriguingly, we can observe a significant sign change from positive to negative correlation for the pair H3K27me3–H3K4me1 and H3K9me3–H3K36me3 between the CpG rich and CpG poor models in HepG2 cells. This suggests that certain epigenetic associated marks are CpG dependent. In fact, it was reported that poised enhancers, which are also marked with H3K4me1 [46], are low in CpG content when they lose H3K27me3 [47].

### Model performance is dependent on HM and CpG content

**Fig 4:**
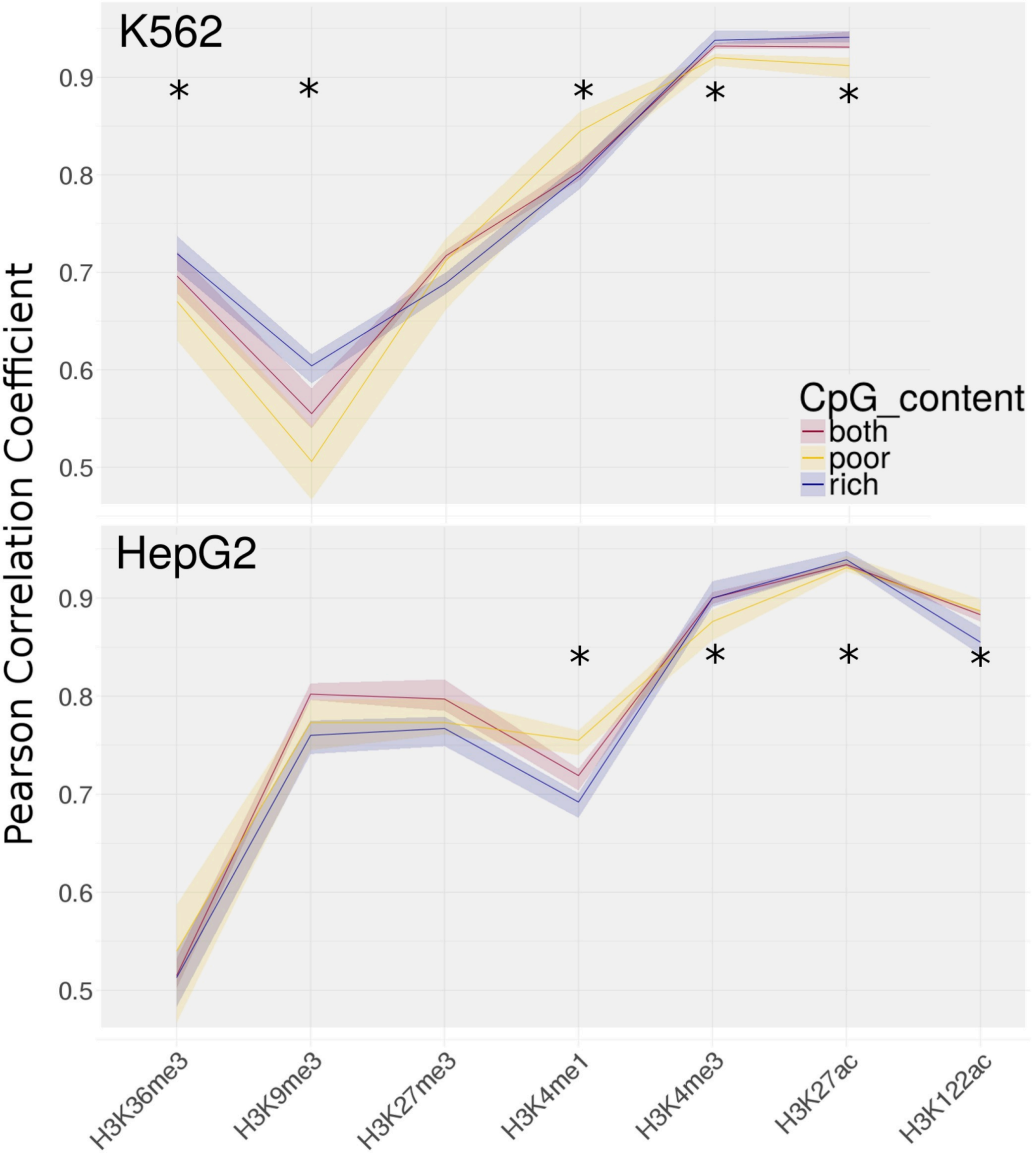
Overall model performance measured as Pearson correlation (y-axis) between predicted and actual response across test sets of outer folds of cross validation for each of the models. Mean correlation coefficient of the 5 folds is indicated by a solid line, range of obtained correlation values across folds is given as the transparent area. We receive one model for each combination of cell line (top plot HepG2, bottom plot K562), CpG type (given by the color encoding), and Histone modification (encoded on the x-axis). Significant differences of performance between CpG rich and poor model according to a Wilcoxon test (*α* = .05, Benjamini-Hochberg corrected) are indicated by asterisks (*).

To infer interactions between TFs and HMs, we trained regularized regression models on TF and HM ChIP-seq data in promoter regions of protein coding genes. We further split the promoters into two classes, high and low CpG content. For each of the models trained, we assessed the individual performance in terms of Pearson correlation between predicted and actual HM abundance. First of all, we can observe that correlation coefficient between model predictions and actual ChIP-seq signals yields different performance trends for individual marks and gene partitions, depicted in Fig 4. In general, we achieve good results with correlation values around 0.9 for the HMs associated with active genes or enhancers, such as H3K27ac and H3K4me3 [29, 46], which meets our expectation that the crosstalk between protein complexes and HMs enable us to predict HM abundance accurately. We can see a drop in performance considering modifications commonly associated with repressed genes, such as H3K27me3 and H3K9me3 [29], with correlation values around 0.8. Both these marks are often found in heterochromatic regions, which also explains the slightly weaker performance of models, as densely packed chromatin presumably reduces DNA accessibility and hence prevents TF binding to a certain extent [48]. Also for H3K36me3, a mark associated with elongation [29], we see that the model does not predict as well as the other marks, with a moderate correlation coefficient of around 0.5.

In addition to comparing models based on the HM’s function, we can contrast them based on the genes’ CpG content. In general, we observe that, in models trained for a given HM, often one of the CpG classes, either models with CpG poor or CpG rich genes, outperforms the respective other class significantly, for instance in all histone marks associated with active genes. To confirm their significance, we performaned a Wilcoxon test with Benjamini-Hochberg test correction (see S5 Table in S1 File) and significance threshold *α* = .05. These results suggest that there is a dependency between TF binding (or their interactions with HMs of interest) and the CpG content. In Fig 4 it also becomes evident that neither CpG rich nor CpG poor genes are dominating the respective other class in all cases in terms of prediction power. Rather, the marks H3K4me3 and H3K27ac, commonly associated with active transcription, are easier to predict with our model in CpG rich genes, but for H3K4me1 the models based on CpG poor genes predict significantly better. Strikingly, the H3K4me1 on the CpG poor genes consistenly outperforms the model with all genes combined in both cell lines.

### Variations in model coefficients corresponding to regions along promoter suggests binding preferences

To get a more fine-grained view on the locality of interactions across the promoter, we split each promoter region into smaller windows (Fig 1), and accumulate ChIP-seq signals in each such window. Although the best models typically show a strong fusion between adjacent windows, that is showing very similar coefficients for adjacent windows, we also observe individual large coefficients for a few windows differing strongly from the small coefficients of the other windows of that promoter. Two interesting cases are visualized in Fig 3. For the TF GATAD2A, we observe higher coefficients in the first three windows compared to the rest in CpG poor models of the H3K27ac and H3K122ac marks. This trend is not evident in the CpG rich models. The TF SAP130 exhibits an exceptionally high coefficient in the last window of CpG rich models, which could be caused by a binding preference of this TF in this region correlated with the HM signals. Furthermore, SAP130 is a sub component of the regulatory complex SIN3A, which shows CG-enriched binding motifs (see S5 Fig in S1 File).

### Inferred associations reflect known interactions and reveal potentially novel interactions

While yielding great performance, it is not given that the models discover biologically meaningful relationships. In this section, we show based on the top 10 statistically significant coefficient of a model that we indeed derive biological relationships that we can validate by literature. We summarized these significant coefficients for HepG2 in Table 1, the corresponding table for K562 can be found in S4 Table in S1 File.

**Table 1:** Overview of the top 10 TFs found significant in HepG2 models. Positive and negative associations, derived from the coefficients, are indicated by red and black dots, respectively.

*HM H3*	K36me3		K9me3		K27me3		K4me1		K4me3		K27ac		K122ac	
*CpG*	+	−	+	−	+	−	+	−	+	−	+	−	+	−
ARID4B			•	•	•	•			•	•		•	•	
CEBPA		•												
DMAP1		•					•		•	•	•		•	•
FOXA3									•					
GABPB1					•				•	•	•			
GATAD1			•	•		•								
GATAD2A	•						•	•		•		•		•
HBP1		•								•				•
HCFC1				•										
HMG20B										•	•			
HOMEZ	•													
KAT8		•		•		•							•	
KDM1A												•		
KLF6									•		•			
KLF16	•								•					
KMT2B	•	•		•	•		•	•			•		•	•
MBD1iso1							•	•						
MBD1iso2					•		•		•	•				
MIER2								•			•	•	•	
MIER3				•										
MXD3	•					•								•
MXD4													•	
NFIL3						•								
NFYC											•		•	•
PAF1	•	•		•		•								
PPARG	•													
RCOR2			•					•						
RFXANK	•													
RXRB			•											•
SAP130			•			•		•	•	•		•		•
SSRP1	•	•	•	•	•	•					•	•		
TBP			•		•		•	•			•	•		•
TEAD3			•	•	•		•	•					•	
TGIF2	•	•						•						
THAP11			•		•		•				•			
SOX5			•											
SP5		•												
SUZ12					•							•		
ZBTB26							•		•	•				•
ZKSCAN8												•		
ZNF48					•	•			•				•	
ZNF511		•				•								
ZNF580							•	•		•				
ZNF644				•								•		
ZNF792													•	

We start with a sanity check to see if we can observe a change from positive to negative correlation for the same TF between an HM that is associated with active transcription and an HM that is linked to repressed genes. Indeed, we can generally observe that the sign of predicted associations of significant TFs changes between models of those different HM classes, e.g. for TBP, SSRP1, TEAD3, or ARID4B. We investigated the biological relevance of predicted associations by exploring the literature, as well as inspecting data of a different experiment, based on ChIP-MS data of mESCs, from a study by Ji et al. found as in [49].

The TATA-binding protein (TBP) is a well known TF linked to transcriptional activation and is crucial for the expression of most genes as a recruiter for RNA Polymerase II [50]. Furthermore, general binding of TBP is facilitated by acetylated histone tails [51], and TBP is known to specifically bind to H3K4me3 [52]. These findings are evident in the ChIP-MS data, where TBP is only abundant in samples of H3K27ac and H3K4me3. In our model TBP is chosen as an important factor with positive association for three models of the acetylated marks and negative association for H3K4me1 models, which is a modification that is mutually exclusive to the H3K4me3 serving as TBP binding site.

The transcription factors ARID4B and SAP130 are both subunits of SIN3A, a co-repressor complex with histone deacetylase (HDAC) activity. More specifically, SIN3A contains HDAC1 and HDAC2 subunits [53], and is found tethered to SET1/ASH2 proteins that trimethylate H3K4 [54]. The relation between SIN3A to the methyltransferase SET1 is reflected in our model, where both ARID4B and SAP130 have positive coefficients in all H3K4me3 models. In the K562 cell line, where SIN3A ChIP-seq data is available, we can also find SIN3A among the top positive associations with H3K4me3 (see S4 Table in S1 File). Interestingly, we find both ARID4B and SAP130 with positive associations in our models for H3K27ac. This is also reflected in the ChIP-MS data, where SIN3A is only detected in samples of the activating marks H3K4me3 and H3K27ac, and not in H3K9me3. A similar case can be found for GATAD2A, which is part of the NuRD complex that contains HDAC1 and HDAC2 as core chromatin modifying components [55]. Our models report positive associations with activating marks such as H3K4me3 and H3K27ac. Again, ChIP-MS data is consistent with our findings, reporting abundance of GATAD2A only in H3K27ac and H3K4me3 samples.

Further examples for the biological relevance of our findings include the Structure Specific Recognition Protein 1 (SSRP1), which is a subunit of the FACT complex, acting as a histone chaperone to reorganize chromosomes to enable transcriptional elongation [56]. H3K27ac, a general activation mark as mentioned before, and H3K36me3, a mark specific for elongation [57], have SSRP1 as one of the top positively associated TFs. Furthermore, all models trained for marks linked to repressive genes have SSRP1 negatively associated to them. The same trend is notable in the ChIP-MS data, where a small amount of the factor can be found for all samples, but for the active marks there is a 3-fold increase in abundance compared to repressive marks. Another case of epigenetic interactions of a protein complex is given by the factor DMAP1 as part of NuA4, a complex with histone acetyltransferase activity reported to have an activating function in transcription [58]. Our models are consistent with this report, showing positive associations between DMAP1 and the activation associated marks H3K27ac and H3K4me3, as well as H3K122ac. Furthermore, DMAP1 is abundant in H3K27ac and H3K4me3 samples in the ChIP-MS data. MIER2, a factor recruiting HDACs [59], has a strong negative association with the HM in models for acetylation marks H3K27ac and H3K122ac.

We can also derive associations with respect to the CpG content classes. For example, THAP11 is associated with binding to HCFC1, a protein complex known to bind to CpG islands and to recruit different chromatin modifying enzymes including KDM1A, a H3K9 demethylase [60]. In our models, THAP11 is suggested as negatively associated to H3K9me3 and H3K27me3 (see Table 1), only for the CpG rich gene set. In models for the CpG poor genes of these two marks, THAP11 shows only a small negative coefficient (see S2 and S3 Figs in S1 File).

As a final example, we examine the relationships of the RNA polymerase II-associated factor 1 homolog (PAF1). This factor interacts with RNA Polymerase II to stimulate transcriptional activity [61]. This behaviour can be observed in our models, where PAF1 is selected with a top positive coefficient in H3K36me3 models of HepG2, and exhibits negative association in models for genes with low CpG content of the repressive marks H3K9me3 and H3K27me3. Furthermore, PAF1 shows a strong positive coefficient in the H3K27ac model for the CpG rich genes of the HepG2 cell line (see S4 Fig in S1 File), even though it is not in the top 10 most significant factors. The ChIP-MS data shows similar results with a high abundance of PAF1 in samples of H3K27ac, H3K4me3, and H3K36me3.

So far, we showed that many of the derived interactions of our models are consistent with reports in the literature as well as results of a ChIP-MS study. This suggests that also novel inferred associations between TFs and HMs reflect biologically meaningful relationships. For instance, the Transcriptional Enhancer Factor TEF-5 (syn. TEAD3) is a protein important for cell growth and tumor suppression [62]. So far, little is known about its epigenetic activity in cells. Our models suggest TEAD3 has a strong negative association with the repressive marks H3K9me3 and H3K27me3, and strong positive association with H3K4me1, which marks enhancers. This points to a possible role of TEAD3 at enhancers, and could be used as a guide for future experimental studies.

### H3K122ac, a marker for active regions

So far, we examined the relationships of TFs with well known HMs and showed that those yield interesting characteristics with regard to CpG content and are biological meaningful. Here, we apply our methodology to H3K122ac—a modification for which the precise function is still unknown—to elucidate its role in gene regulation. First studies suggest that this mark, located at the dyad axis of the nucleosome, destabilizes the nucleosome complex, thus loosening up densely packed chromatin and stimulating transcription [37]. Our models were are able to predict abundance of H3K122ac nearly as accurate as the other modifications linked to active genes, with correlations between 0.85 and 0.9 across the different models as illustrated in Fig 4. We discover hints for an association with activation when comparing the most significant TFs found for the different HMs in the HepG2 cell line, with similar TFs picked in H3K122ac and the marks H3K27ac and H3K4me3 associated with active genes (Table 1). Also, important TFs such as TBP, SAP130, or KMT2B have opposing signs between H3K122ac and the models of the H3K9me3 and H3K27me3, which are linked to repressed genes. Furthermore, TBP, a tightly regulated TF crucial for the initiation and activation of transcription for most genes [50], shows the highest coefficient in the H3K122ac model for CpG poor genes, further supporting the idea of H3K122ac to be linked to active transcription.

However, the partial correlation study (Fig 3) indicates a strong positive partial correlation between H3K122ac and H3K27ac, and a strong negative partial correlation between H3K122ac and H3K9me3. On the one hand, this further supports the hypothesis of H3K122ac being a mark commonly associated with active transcription. On the other hand, this shows that the co-occurrence of H3K122ac with H3K27ac could also lead to similar TFs being picked for both models, with one of the HMs acting as a confounder for the other HM.

## Discussion

In our analysis, we show that with the suggested models we were able to accurately predict histone modifications based on TF abundance and thereby infer biologically meaningful relationships. We observe a difference between models trained on modifications that are linked to active genes, such as H3K27ac and H3K4me3, performing better than models of marks that are mostly associated with repressed genes, such as H3K9m3 and H3K27me3. One reason could be that the latter are reported as marks for heterochromatic regions, densely packed chromatin, where it is in general more difficult for TFs to bind as most of the DNA is not directly accessible. Thus, there is less information available to the model, because our input is TF ChIP-seq measurements. We also observe a drop in performance when looking at H3K36me3 compared to other models. This mark is an elongation mark and as such, it is mostly found in the gene body [63], whereas we are interested in HM presence at the vicinity of the transcription start site. Besides, there is also a difference between CpG rich and CpG poor model performance evident throughout all models, but we observe that for certain marks the CpG poor model performs better and for others the CpG rich model performance is superior. It is therefore unlikely that the differences are due to technical biases based on the CpG content of the sequences, but rather reflect actual CpG dependent associations. Moreover, we observe a discrepancy in the performance of the HepG2 and the K562 cell lines. There are several reasons that could lead to this discrepancy in the performance, for instance different expression patterns between these two cell lines. These differences in gene expression based on cell type specific regulatory programs also involve a different epigenetic landscape and hence HM and TF distribution, which may lead to different associations being found. Also available experimental measurements are distinct between the two cell lines, with certain TFs being only measured for one of the cell lines. Additionally, the observed difference for the H3k36me3 mark between the two cell lines is likely due to its role in transcriptional elongation as mentioned above.

Within the scope of the literature search to assess biological relevance of the found associations, we discovered subtle relationships that could not be explained by individual literature alone. For example, ARID4B and SAP130 are both subunits of the SIN3A complex, which is involved in histone deacetylation [53]. However, these TFs are found to be associated with H3K4me3 and H3K27ac, which are linked to active promoter regions. Both these associations were also present in ChIP-MS data of mESCs of the study of Ji et al. [49], where both H3K4me3 and H3K27ac were co-located with these proteins. The link to H3K4me3 could be explained by the physical interaction of the SIN3A complex with SET1/ASH2 [54], which are proteins that trimethylate H3K4. The association with H3K27ac seems to contradict the histone deacetylase activity of the SIN3A complex. Recently however, an ongoing dynamic turnover of acetylation, crucial for the orchestration of gene expression, was hypothesized and supported by experimental results [64–66]. Assuming this rapid dynamic turnover combined with the fact that SIN3A establishes H3K4me3 modification, one hypothesis is that the traditional view of SIN3A being a repressor is too generic, but it rather acts as a complex that tightly regulates transcription by dynamic activation and repression. Another hypothesis would be that the occurrence of H3K27ac as important association is due to H3K4me3 acting as confounding variable. These two marks seem to be often co-localized, which was further supported by our partial correlation study.

We also find that certain TFs, such as GATAD2A show binding preferences depending on the CpG content. For GATAD2A, there seems to be a binding preference indicated by high coefficients for certain bins of the promoter window for H3K27ac and H3K122ac. There is no such trend apparent in the CpG rich models of marks associated with transcriptional activation, where this TF is also generally not picked as an important feature of the model (compare Table 1). This suggests that GATAD2A interaction with HMs is dependent on the CpG content of the promoter region. In fact, GATAD2A is a structural component of the NuRD complex, which binds to methylated cytosines of the DNA through MBD2 [67, 68]. This could explain the absence of GATAD2A as an important feature for CpG rich models, as CpG islands are predominantly unmethylated [69] and MBD2 shows a CG enriched binding motif (see S6 Fig in S1 File).

## Conclusion

In this work we extended existing approaches that investigate the relationship between TFs and HMs in the light of gene regulation based on ChIP-seq data. In particular, we studied TF–HM relationships in the context of sequence CpG content and expand the work to the H3K122ac mark, about little has been known so far. We use the regularized regression method Fused LASSO [39] to predict the abundance of HMs in promoter regions based on the abundance of a set of TFs in these regions. We leverage the coefficients of each model as a mean to measure positive as well as negative associations between TFs and HMs. One drawback of this approach is the limitation to linear models, which are not able to discover nonlinear associations. However, nonlinear models are hard to interpret and are thus less not suited for exploratory studies as this one. We complemented the regression models with a partial correlation study of the considered HMs in the context of sequence CpG content and further make use of these results in the interpretation of the regression models to avoid inferring confounded associations. Here, we gathered data for K562 and HepG2 cell lines using existing ChIP-seq measurements of a large set of TFs from ENCODE [4]. The ChIP-seq measurements of the HMs H3K27ac, H3K4me1, H3K4me3, HeK9me3, H3K27me3, H3K36me3 for K562 were obtained from the ENCODE database. The same HMs and additionally H3K122ac were measured through ChIP-seq for the HepG2 cells as part of the DEEP project [6, 44].

For the modeling, we complement the method of Benveniste et al. [34] by using a regression approach instead of classification. Thus, we are also able to capture negative association and implicitly obtain a measure of association strength, which binary classification can not handle. Compared to Perner et al. [20] we do not limit the features only to known chromatin modifiers, but also incorporate other TFs that allow us to derive potential novel associations. In contrast to both methods we use a higher resolution of the TFs by partitioning the promoter regions into small bins. Our main contribution is the extension of the association study by separating the set of promoters into CpG rich and CpG poor promoters, to investigate their impact on the relationship between TFs and HMs. We explored the relationship between TFs and H3K122ac in HepG2 cells, a HM about little is known so far, suggesting TFs involved in its regulation by investigating model coefficients and taking the partial correlation with the other HMs into account.

To assess the biological relevance of the discovered associations, we additionally searched the literature for the results of the HepG2 cell line. We were able to explain many of the derived relationships directly by, for instance, interaction of TFs with a chromatin modifying complex, or TFs building a subunit of such a complex. Several other discovered associations could be explained by reportedly strong connections of the TF with active transcription, where the positively associated marks were linked to active transcription and negatively associated marks to repressed transcription. We also found relationships that were more subtle, such as ARID4B and SAP130 associated with H3K27ac, leaving room for speculation. The results of our model can also be used to guide future work on discovering novel interaction partners between TFs and HMs, as some of the discovered associations have not been reported in the literature and thus could serve as a starting point for interaction studies.

As a new result, we investigated the influence of CpG content on the prediction performance and discovered associations. We found that the model performance is dependent on the CpG content, with the CpG rich models being superior to the CpG poor models for H3K4me3 and H3K27ac, but inferior for the H3K4me1 models. Moreover, we discovered that certain TFs can only be found as important and significant in one of the two groups, such as THAP11. This suggests that CpG content is a key factor for TF-HM relationships and thus should be incorporated in studies of TF and HM data.

To summarize, we showed that our regularized regression approach performs well and was able to recover known positive as well as negative associations between TFs and HMs and thus could be used to guide future search for novel interactions. Furthermore, we found that the CpG content has an impact on regression performance, which could be explained by the the binding preference of certain TFs to only CpG poor respectively rich genes. As only little research has focused on this dependence, we were only able to back up the findings for THAP11 by literature. Nonetheless, these results indicate that the CpG content is an important factor in the gene regulatory system and its information content should be leveraged in computational models, if possible. Finally, our investigation of H3K122ac suggests a role as a mark for active regions, which is not only evident in the regression models but is also supported by the partial correlation study.

## Supporting information

S1 File (PDF)
