# Supplementary material for: CpG content-dependent associations between transcription factors and histone modifications
Source: PLoS One. 2021 Apr 15;16(4):e0249985. doi: 10.1371/journal.pone.0249985 (PMC8049299; doi:10.1371/journal.pone.0249985)
Supplement: S1 File — (PDF) [file pone.0249985.s001.pdf]

S1 File.
CpG content-dependent associations between transcription factors and histone modifications

Jonas Fischer, Fatemeh Behjati Ardakani, Kathrin Kattler,
Jörn Walter, Marcel H. Schulz

March 10, 2021

1 Supplementary Figures

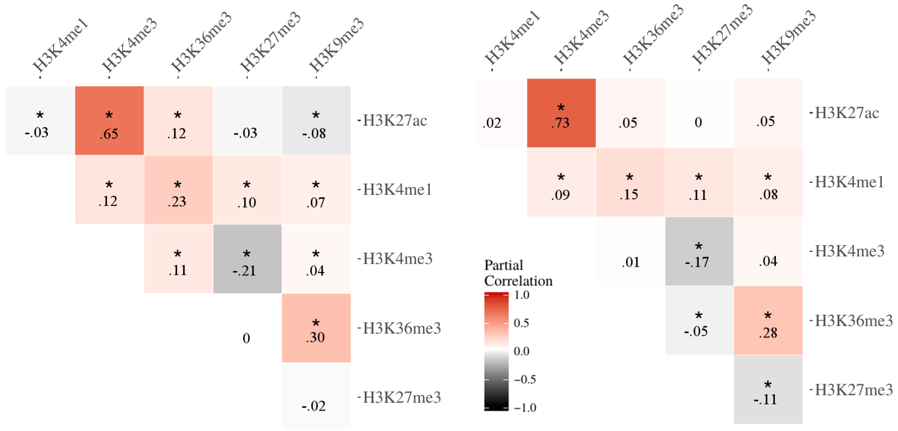

S1 Fig: *Partial correlation for K562* Partial correlation heatmap between Histone Modifications in CpG rich (left) and poor (right) promoters in K562. Partial correlation coefficients are computed on mean ChIP-seq signal on the promoter regions of the gene sets. Significant partial correlations are marked with $*$.

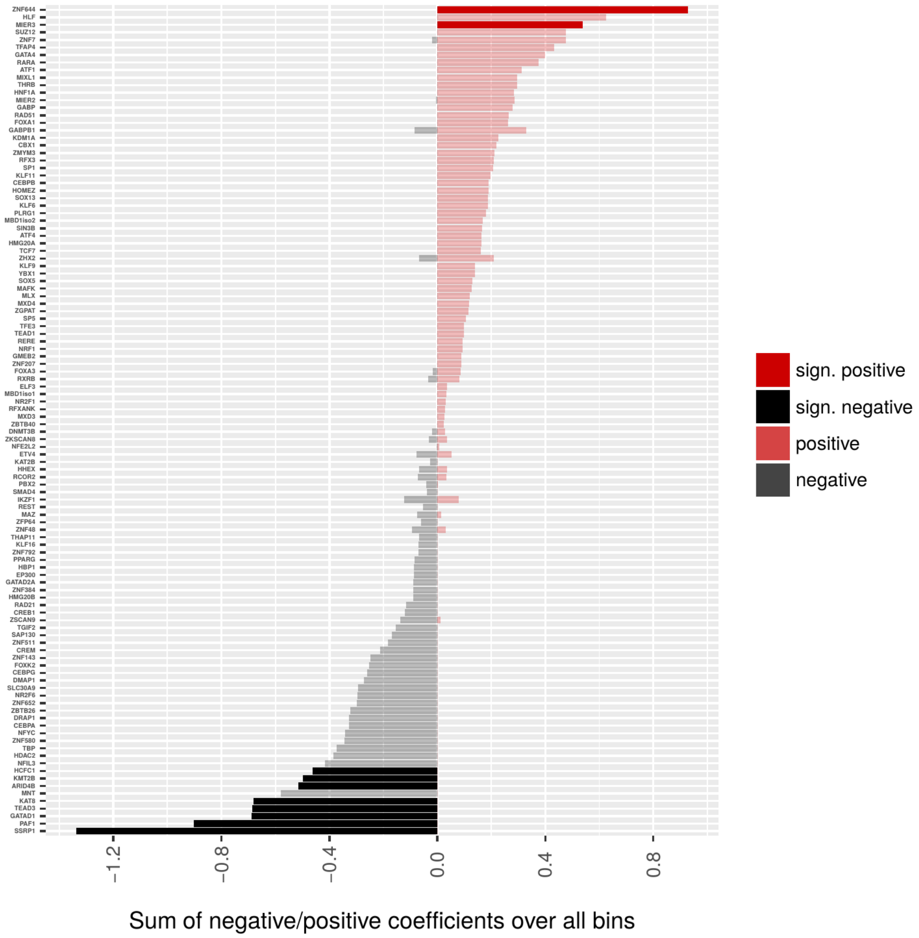

S2 Fig: *Model coefficient summary for H3K9me3 in CpG poor genes of HepG2.* For each TF (x-axis), the sum over the negative and positive coefficients across bins are indicated by black and red bars, respectively. TFs that are not under the top 10 significant TFs according to an F-test are transparent.

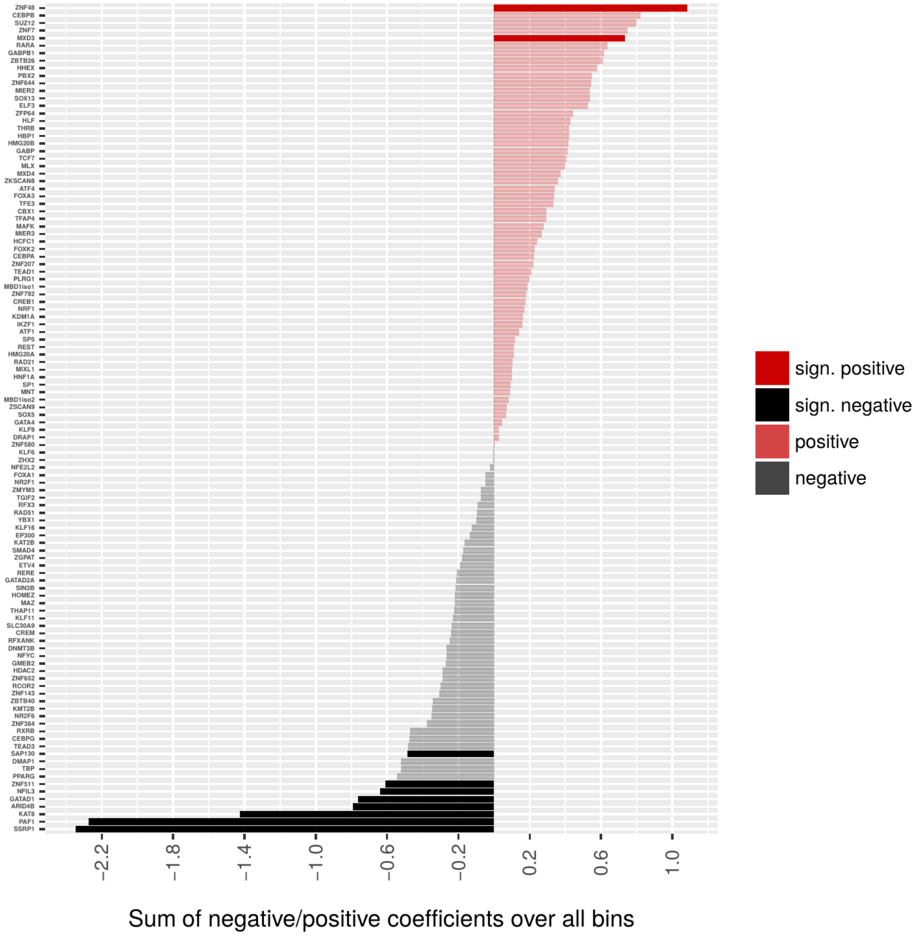

S3 Fig: *Model coefficient summary for H3K27me3 in CpG poor genes of HepG2.* For each TF (x-axis), the sum over the negative and positive coefficients across bins are indicated by black and red bars, respectively. TFs that are not under the top 10 significant TFs according to an F-test are transparent.

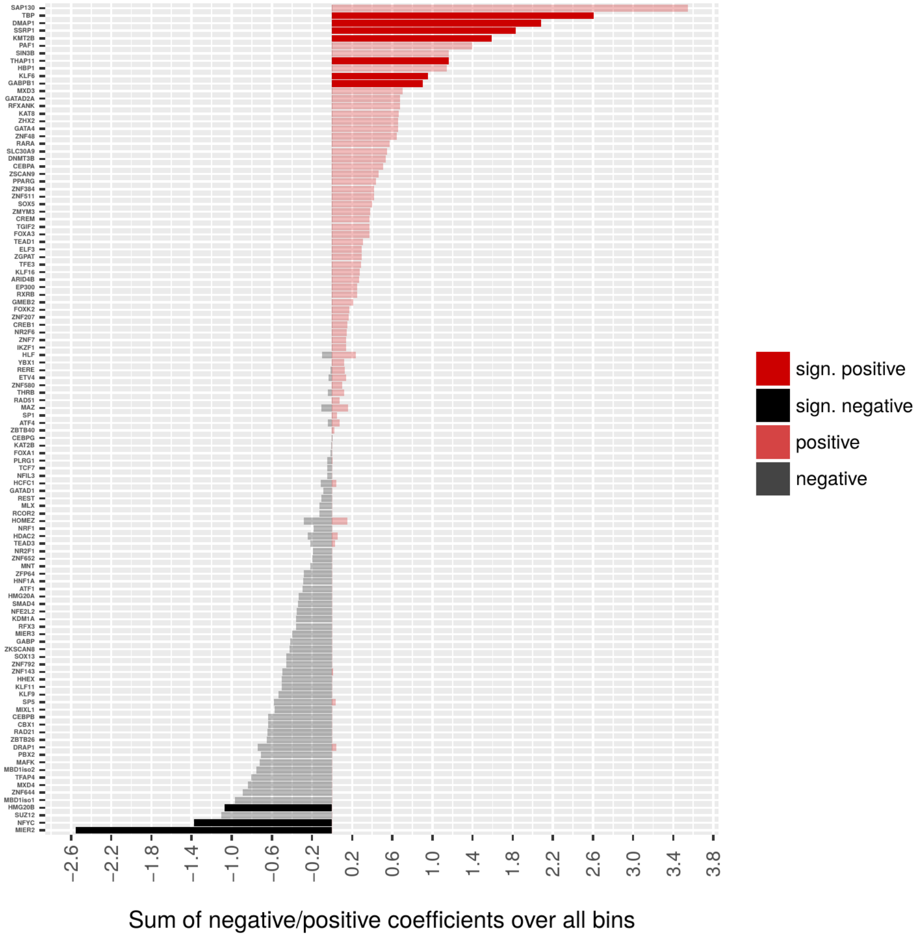

S4 Fig: *Model coefficient summary for H3K27ac in CpG rich genes of HepG2.* For each TF (x-axis), the sum over the negative and positive coefficients across bins are indicated by black and red bars, respectively. TFs that are not under the top 10 significant TFs according to an F-test are transparent.

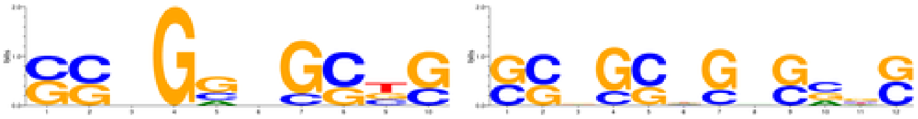

S5 Fig: *SIN3A binding motifs* Visualized are two of the top ten binding motifs for SIN3A taken from the Kellis database [1].

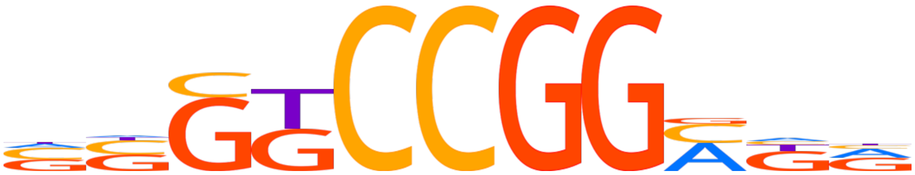

S6 Fig: *MBD2 binding motif* Visualized is the binding motif of NuRD subcomponent MBD2 as given by HOCOMOCO [2].

2 Supplementary Tables

S1 Table: K562 ENCODE Transcription Factor Experiment IDs

TF	ExperimentID	TF	ExperimentID
ADNP	ENCFF946EOR	MYNN	ENCFF145XHA
ARID1B	ENCFF750FNO	NBN	ENCFF676AOK
ARNT	ENCFF421DLL	NCOA1	ENCFF941LGJ
ATF1	ENCFF829DDT	NCOR1	ENCFF211OZC
ATF3	ENCFF175VPS	NFE2	ENCFF670JFE
BACH1	ENCFF968KJF	NFRKB	ENCFF231QPU
BCLAF1	ENCFF626JTH	NFXL1	ENCFF906FIL
BCOR	ENCFF243LGY	NR2C2	ENCFF657ZPH
BHLHE40	ENCFF765OJY	NR2F1	ENCFF928QBF
BMI1	ENCFF424UYP	NR2F6	ENCFF142EXA
BRD4	ENCFF260JHC	NR4A1	ENCFF040AXK
C11orf30	ENCFF219HQE	NRF1	ENCFF720BUE
CBX1	ENCFF836DWS	PBX2	ENCFF840IZZ
CBX3	ENCFF630YDI	PKNOX1	ENCFF834YRR
CBX5	ENCFF014XHT	PTRF	ENCFF201WOP
CDC5L	ENCFF739MHD	PTTG1	ENCFF997ETO
CEBPB	ENCFF212NWU	PYGO2	ENCFF056OBU
CEBPG	ENCFF481CQW	RAD51	ENCFF532QDR
CHAMP1	ENCFF087XWL	RELA	ENCFF475GOZ
COPS2	ENCFF684JZK	REST	ENCFF571ECM
CREB3	ENCFF879MML	RFX1	ENCFF362QFP
CREB3L1	ENCFF010MNE	RING1	ENCFF935ISX
CUX1	ENCFF769QZE	RNF2	ENCFF320VKN
DDX20	ENCFF571WII	RUNX1	ENCFF962DIA
DEAF1	ENCFF187GTP	SIN3A	ENCFF783JQV
DIDO1	ENCFF780XYT	SIX5	ENCFF925ZXV
DNMT1	ENCFF820AVX	SMAD2	ENCFF437PDZ
DPF2	ENCFF950HQL	SMAD5	ENCFF024UMO
DROSHA	ENCFF147MQS	SMARCA4	ENCFF748JPG
E2F5	ENCFF672OUR	SMARCA5	ENCFF847GKQ
ELF1	ENCFF439ZHX	SMARCE1	ENCFF072YCS
ELK1	ENCFF060BLM	SP1	ENCFF858NSY
ESRRA	ENCFF827COK	SREBF1	ENCFF063EPQ
ETS2	ENCFF513JDH	STAT1	ENCFF785LIV
ETV1	ENCFF283MZY	STAT2	ENCFF133YDB
ETV6	ENCFF884NXM	SUPT5H	ENCFF754ZCQ

FOSL1	ENCFF554DIU	SUZ12	ENCFF363DWX
FOXA1	ENCFF147BRO	TAF7	ENCFF926TKQ
FOXJ2	ENCFF658PJX	TARDBP	ENCFF853HNW
FOXK2	ENCFF852WRN	TBL1XR1	ENCFF350TPD
GABPA	ENCFF915JOK	TCF7	ENCFF580KAN
GATA1	ENCFF226FPS	TCF7L2	ENCFF899DYD
GATA2	ENCFF148KWA	TEAD2	ENCFF048PQD
GTF2A2	ENCFF741INH	TFDP1	ENCFF462NRC
GTF2E2	ENCFF974CYT	THRAP3	ENCFF341OHT
HCFC1	ENCFF633TLX	TRIM24	ENCFF868YDK
HDAC1	ENCFF645PUW	TRIM28	ENCFF910BQM
HDAC2	ENCFF697WDH	TSC22D4	ENCFF380LKG
HDAC8	ENCFF156FQS	USF2	ENCFF585WCX
HDGF	ENCFF224ASU	YBX1	ENCFF430MEW
HES1	ENCFF071USV	YBX3	ENCFF889PGD
HINFP	ENCFF129FNZ	ZBED1	ENCFF557XQA
HMBOX1	ENCFF614EKZ	ZBTB11	ENCFF628WOU
ID3	ENCFF367HWH	ZBTB2	ENCFF715FKT
IKZF1	ENCFF715OCK	ZBTB40	ENCFF882ZUY
ILK	ENCFF192BQU	ZBTB7A	ENCFF457PSO
IRF1	ENCFF979SUZ	ZEB2	ENCFF120VOJ
IRF9	ENCFF482EGT	ZFX	ENCFF962YAA
JUNB	ENCFF511DBK	ZKSCAN1	ENCFF181YKE
JUND	ENCFF709JGL	ZKSCAN8	ENCFF029MZM
JUN	ENCFF506EKM	ZMIZ1	ENCFF257YKB
KAT8	ENCFF068JOG	ZMYM3	ENCFF711IOY
KDM1A	ENCFF758MEL	ZNF175	ENCFF869FIE
KDM4B	ENCFF628CJE	ZNF197	ENCFF496HOU
KLF13	ENCFF229SYD	ZNF24	ENCFF350TWP
KLF16	ENCFF161TZI	ZNF316	ENCFF617XZE
KLF1	ENCFF061GJT	ZNF318	ENCFF609CBO
L3MBTL2	ENCFF127DIL	ZNF407	ENCFF972MUU
MAFG	ENCFF035CJL	ZNF512	ENCFF870WHI
MAFK	ENCFF964KPV	ZNF584	ENCFF819OJE
MCM3	ENCFF332WPW	ZNF589	ENCFF784XPD
MCM5	ENCFF243CWO	ZNF639	ENCFF312PLO
MCM7	ENCFF028PQJ	ZNF644	ENCFF908OPJ
MEF2D	ENCFF311QYP	ZNF740	ENCFF621LZE
MGA	ENCFF932XMO	ZNF766	ENCFF059DNT
MIER1	ENCFF332DMO	ZNF83	ENCFF433QCW
MITF	ENCFF427TQP	ZSCAN29	ENCFF935GAH

MLLT1	ENCFF330XNK	ZZZ3	ENCFF716WJE
MTA2	ENCFF653CUO		
MYC	ENCFF174LWV		

S2 Table: HepG2 ENCODE Transcription Factor Experiment IDs

TF	ExperimentID	TF	ExperimentID
ARID4B	ENCFF154HFK	NR2F1	ENCFF322YEC
ATF1	ENCFF368XHC	NR2F6	ENCFF351HQV
ATF4	ENCFF661GPF	NRF1	ENCFF168SMR
CBX1	ENCFF125CDK	PAF1	ENCFF830ZNT
CEBPA	ENCFF087AEC	PBX2	ENCFF363DJS
CEBPB	ENCFF812DOO	PLRG1	ENCFF626AUN
CEBPG	ENCFF668EYP	PPARG	ENCFF228JSX
CREB1	ENCFF664TLP	RAD21	ENCFF120BII
CREM	ENCFF748IWJ	RAD51	ENCFF773TRV
DMAP1	ENCFF539XWR	RARA	ENCFF664JQM
DNMT3B	ENCFF837AEU	RCOR2	ENCFF952ZYO
DRAP1	ENCFF026EFC	RERE	ENCFF402UZM
ELF3	ENCFF172EWV	REST	ENCFF843JOP
EP300	ENCFF632FVW	RFX3	ENCFF227UAR
ETV4	ENCFF574GHX	RFXANK	ENCFF817JXD
FOXA1	ENCFF923YCX	RXRB	ENCFF083BFM
FOXA3	ENCFF038AIJ	SAP130	ENCFF846DFV
FOXK2	ENCFF037PRK	SIN3B	ENCFF875SAX
GABPB1	ENCFF613JIZ	SLC30A9	ENCFF731PDO
GABP	ENCFF215XMA	SMAD4	ENCFF916ADP
GATA4	ENCFF208YII	SOX13	ENCFF396ARC
GATAD1	ENCFF941UWE	SOX5	ENCFF506FGX
GATAD2A	ENCFF890FKS	SP1	ENCFF732DBE
GMEB2	ENCFF164AVP	SP5	ENCFF114DWU
HBP1	ENCFF977MSQ	SSRP1	ENCFF043OFA
HCFC1	ENCFF300UHW	SUZ12	ENCFF406ZOC
HDAC2	ENCFF520QJN	TBP	ENCFF422EPR
HHEX	ENCFF970TCG	TCF7	ENCFF803QZF
HLF	ENCFF652BFV	TEAD1	ENCFF602IQI
HMG20A	ENCFF903JLK	TEAD3	ENCFF322HKP
HMG20B	ENCFF714ZPK		
HNF1A	ENCFF104YZH	TFAP4	ENCFF422FDS
HOMEZ	ENCFF991GWU	TFE3	ENCFF096WTD
IKZF1	ENCFF284DOX	TGIF2	ENCFF345LNB
KAT2B	ENCFF306HMD	THAP11	ENCFF363YQT

KAT8	ENCFF423MHO	THRB	ENCFF025ZTI
KDM1A	ENCFF617NUG	YBX1	ENCFF681OZI
KLF11	ENCFF607AJC	ZBTB26	ENCFF646IGY
KLF16	ENCFF429LCD	ZBTB40	ENCFF557NCF
KLF6	ENCFF017BUZ	ZFP64	ENCFF251OUD
KLF9	ENCFF401LXI	ZGPAT	ENCFF423DKP
KMT2B	ENCFF230IJN	ZHX2	ENCFF517YTG
MAFK	ENCFF816KVE	ZKSCAN8	ENCFF684MOO
MAZ	ENCFF509RVQ	ZMYM3	ENCFF494NAX
MBD1iso1	ENCFF176TOU	ZNF143	ENCFF268KVM
MBD1iso2	ENCFF010ZCB	ZNF207	ENCFF293HMA
MIER2	ENCFF214FNF	ZNF384	ENCFF385RJP
MIER3	ENCFF398CCH	ZNF48	ENCFF674SCB
MIXL1	ENCFF697FMA	ZNF511	ENCFF136GRX
MLX	ENCFF276RTX	ZNF580	ENCFF392EGH
MNT	ENCFF629MWM	ZNF644	ENCFF649PVC
MXD3	ENCFF897JCJ	ZNF652	ENCFF509VMF
MXD4	ENCFF375KVX	ZNF792	ENCFF220ZAV
NFE2L2	ENCFF645MZA	ZNF7	ENCFF486XEI
NFIL3	ENCFF142JQU	ZSCAN9	ENCFF874XIR
NFYC	ENCFF471DGZ		

Histone Mod.	K562
H3K27ac	ENCSR000AKP
H3K27me3	ENCSR000AKQ
H3K4me1	ENCSR000AKS
H3K4me3	ENCSR000AKU
H3K9me3	ENCSR000APE
H3K36me3	ENCSR000AKR

S3 Table: *K562 HM accession IDS.* Accession IDs for ENCODE for HM data used for K562 models.

HM	H3K36me3		H3K9me3		H3K27me3		H3K4me1		H3K4me3		H3K27ac	
CpG	+	−	+	−	+	−	+	−	+	−	+	−
ARID1B	● black		● black									
ATF1										● red		
BCOR		● black						● black				
BHLHE40							● red					
BMI1					● red	● red						
BRD4											● red	● red
CBX1	● black	● black	● red	● red							● black	● black
CBX3			● red	● red	● black	● black	● red	● red				● red
CBX5			● red	● red								
CHAMP1			● red									
CREB3L1										● red		
DPF2							● red	● red				
E2F5				● black								
GATA2		● red										
HCFC1	● black				● black		● black		● red			
HDAC1						● red			● red	● red		
HDGF	● red	● red				● black	● red	● red				
ID3								● red				
JUND			● black	● black			● red		● red		● red	
KDM4B				● black	● red							
L3MBTL2		● black					● red		● red	● red		
MCM3		● black				● red						
MCM7						● red						
MGA									● black			
MLLT1								● black				
MTA2			● black								● black	
MYNN											● black	● black
NFRKB		● black									● red	● red
NR4A1										● red		
RING1												● black
RNF2					● red	● red			● red	● red		
SIN3A	● red				● black				● red	● red	● red	● red
SMAD5								● black	● red	● red	● red	● red
SMARCA4								● red		● red		
SMARCE1								● red				
SP1	● black											
SREBF1	● red	● red					● red	● red				
SUPT5H	● red	● red		● black	● black	● black					● red	● red
SUZ12					● red	● red						
TARDBP				● black		● red						
TBL1XR1	● red		● red		● red		● red					
TCF7			● black									
TRIM24									● black			
ZBTB7A	● black	● black	● black	● black								
ZBTB40					● black		● red		● red	● red		● red
ZNF639				● black								
ZKSCAN1											● red	

S4 Table: Overview of the top 10 TFs found significant in K562 models. Positive and negative associations, derived from the coefficients, are indicated by red and black dots, respectively.

	p-value	
HM	K562	HepG2
H3K4me1	0.0129	0.0129
H3K4me3	0.0129	0.0129
H3K9me3	0.0129	0.0657
H3K27me3	0.0657	0.6905
H3K27ac	0.0129	0.0230
H3K36me3	0.0129	0.1634
H3K122ac	—	0.0129

S5 Table: Bejamini-Hochberg corrected p-values according to Wilcoxon tests performend between CpG-rich and poor model performances. The tests are computed on spearman correlations on the 5 outer folds of the cross validation.

References

[1] P. Kheradpour and M. Kellis. Systematic discovery and characterization of regulatory motifs in ENCODE TF binding experiments. *Nucleic Acids Res.*, 42(5):2976–2987, 2014.

[2] I. V. Kulakovskiy, Y. A. Medvedeva, U. Schaefer, A. S. Kasianov, I. E. Vorontsov, V. B. Bajic, and V. J. Makeev. HOCOMOCO: towards a complete collection of transcription factor binding models for human and mouse via large-scale ChIP-Seq analysis. *Nucleic Acids Res.*, 46(D1):D252–D259, 2018.
